# Supplementary material for: Determinants of evidence-based practice among health care professionals in Ethiopia: A systematic review and meta-analysis
Source: PLoS One. 2023 Nov 9;18(11):e0293902. doi: 10.1371/journal.pone.0293902 (PMC10635493; doi:10.1371/journal.pone.0293902)
Supplement: S2 Table — (DOCX) [file pone.0293902.s002.docx]

| **Supplementary table 2 :** Newcastle-Ottawa Quality Assessment Scale for cross sectional studies used in the systematic review and meta-analysis 2022 | | | | | | | | |
| --- | --- | --- | --- | --- | --- | --- | --- | --- |
|  | Selection | | | | Comparability | Outcome | | Total score |
| Authors | Representativeness s (1) | Sample size (1) | Non respondents (1) | Ascertainment of the exposure (risk factor) (2) | The subjects in different outcome groups are comparable, based on the study design or analysis. confounding factors are controlled (2) | Assessment of the outcome (2) | Statistical test (1) |  |
| Alemayehu, et al. | 1 | 1 | 1 | 1 | 1 | 2 | 1 | 8 |
| Wurjine, , et al. | 1 | 1 | 1 | 2 | 1 | 1 | 1 | 8 |
| Abera, et al. | 1 | 1 | 1 | 2 | 1 | 1 | 1 | 8 |
| Dereje, et al. | 1 | 1 | 1 | 2 | 1 | 2 | 1 | 9 |
| Hoyiso, et al. | 1 | 1 | 1 | 2 | 1 | 2 | 1 | 9 |
| Megersa, et al. | 1 | 1 | 1 | 1 | 1 | 2 | 1 | 8 |
| Wodajo, et al. | 1 | 1 | 1 | 2 | 1 | 1 | 1 | 8 |
| Worku, et al. | 1 | 1 | 1 | 1 | 1 | 2 | 1 | 8 |
| Alene, et al. | 1 | 1 | 1 | 2 | 1 | 1 | 1 | 8 |
| Assefa, et al. | 1 | 1 | 1 | 2 | 1 | 1 | 1 | 8 |
| Hadgu, et al. | 1 | 1 | 1 | 2 | 1 | 2 | 1 | 9 |
| Mititku, et al. | 1 | 1 | 1 | 2 | 1 | 2 | 1 | 9 |
| Aynalem, et al. | 1 | 1 | 1 | 1 | 1 | 2 | 1 | 8 |
| Beshir, et al. | 1 | 1 | 1 | 2 | 1 | 1 | 1 | 8 |
| Dagne, et al. | 1 | 1 | 1 | 1 | 1 | 2 | 1 | 8 |
| Debeb, et al. | 1 | 1 | 1 | 2 | 1 | 1 | 1 | 8 |
| Degu, et al. | 1 | 1 | 1 | 2 | 1 | 1 | 1 | 8 |
| Dessie, et al. | 1 | 1 | 1 | 2 | 1 | 2 | 1 | 9 |
| Kassahun, et al. | 1 | 1 | 1 | 2 | 1 | 2 | 1 | 9 |
| Melesew, et al. | 1 | 1 | 1 | 1 | 1 | 2 | 1 | 8 |
| Wassie, et al. | 1 | 1 | 1 | 2 | 1 | 1 | 1 | 8 |
| Yehualashet,et al. | 1 | 1 | 1 | 1 | 1 | 2 | 1 | 8 |
| Yideg, et al. | 1 | 1 | 1 | 2 | 1 | 1 | 1 | 8 |
