# Supplementary material for: Determinants of evidence-based practice among health care professionals in Ethiopia: A systematic review and meta-analysis
Source: PLoS One. 2023 Nov 9;18(11):e0293902. doi: 10.1371/journal.pone.0293902 (PMC10635493; doi:10.1371/journal.pone.0293902)
Supplement: Attachment — Submitted filename: Review on EBP.docx [file pone.0293902.s003.docx]

This submission presents the results of determinants of evidence-based practice utilization among health care professionals in Ethiopia: a systematic review and meta-analysis. The research question was interesting and has public health relevance even though I have some queries regarding the manuscript.

Specific comments/questions are listed below.

1. In affilation number 2 the word “Woldia” was not correctly spelled.
2. The whole text needs to be written in correct English, to prevent misunderstanding.
3. I think the title should be re-written again since the use of words like “ *practice*” and “*utilization*” consequatively is ambigious.
4. The last statement of your conclusion in the abstract should be re-written.
5. In your discussion of the pooled prevalence you have reason out the *availability teaching curriculum of health professionals in Nigeria and Uganda* increases the EBP; what is the authors' reference for this?
6. “*The finding suggest future intervention for improving evidence based medicine should focus on addressing those organizational barriers through availing standard guideline at work place, accessing internet in the work environment and minimizing professional workload that helps them to critically appraise and integrate latest research evidence for clinical decision making.”* This statement at the end of the discussion should not be the part of the abstract.
