# Supplementary material for: Determinants of evidence-based practice among health care professionals in Ethiopia: A systematic review and meta-analysis
Source: PLoS One. 2023 Nov 9;18(11):e0293902. doi: 10.1371/journal.pone.0293902 (PMC10635493; doi:10.1371/journal.pone.0293902)
Supplement: Attachment — Submitted filename: Response to Reviewers.edited.edited.docx [file pone.0293902.s004.docx]

**A point-by-point response to reviewers and editor**

First of all, we would like to say thank you to both the reviewers and editor, we are so lucky to have you who had a great contribution to the improvement of this work. Saying this we have addressed the given comments as follows.

**Reviewer #2**

**Comment:** In affilation number 2 the word “Woldia” was not correctly spelled.

**Response:** ok thank you for your comment. We would like to say sorry for the spelling error we have made in the institution naming. We accept the reviewer's comment and we will correct it in the revised manuscript as shown in the track change.

**Comment:**  The whole text needs to be written in correct English, to prevent misunderstanding.

**Response:** ok thank you for your comment. We accept the reviewer's comment and try to edit the revised document by consulting an English-proficient individual to avoid misunderstandings that can arise from grammar, punctuation, and spelling errors.

**Comment:** I think the title should be re-written again since the use of words like “ *practice*” and “*utilization*” consequatively is ambigious.

**Response:** ok thank you for your comment. We accept your comment and rewrite it as shown in the track change of the revised manuscript.

**Comment:**  The last statement of your conclusion in the abstract should be re-written.

**Response:** ok thank you for your comment. We accept your comment and rewrite it as shown in the track change of the revised manuscript.

**Comment:** In your discussion of the pooled prevalence you have reason out the *availability teaching curriculum of health professionals in Nigeria and Uganda* increases the EBP; what is the authors' reference for this?

**Response:** ok thank you for the question raised. Our justification for lower EBP in Ethiopia as compared to *health professionals in Nigeria and Uganda is* the difference in EBP awareness and integration of EBP in the teaching curriculum of health professionals. Our reference for this is the cited article in the comparison. While we read the cited article it says most (90.2) of the respondents have good knowledge about evidence-based practice that might be acquired through curriculum-based training in their formal education.

**Comment:** “*The finding suggest future intervention for improving evidence based medicine should focus on addressing those organizational barriers through availing standard guideline at work place, accessing internet in the work environment and minimizing professional workload that helps them to critically appraise and integrate latest research evidence for clinical decision making.”* This statement at the end of the discussion should not be the part of the abstract.

**Response:** ok thank you for your comment we accept the reviewer's suggestion and we will put it in the abstract in the conclusion subsection as you suggested above in the revised manuscript as shown in the track change.
