# Supplementary material for: Determinants of evidence-based practice among health care professionals in Ethiopia: A systematic review and meta-analysis
Source: PLoS One. 2023 Nov 9;18(11):e0293902. doi: 10.1371/journal.pone.0293902 (PMC10635493; doi:10.1371/journal.pone.0293902)
Supplement: Attachment — Submitted filename: Response to Reviewers.2nd.docx [file pone.0293902.s005.docx]

**A point-by-point response to reviewers and editor**

First of all, we would like to say thank you to both the reviewers and editor, we are so lucky to have you who had a great contribution to the improvement of this work. Saying this we have addressed the given comments as follows.

**Journal requirements**

please review your reference list to ensure that it is complete and correct. If you have cited papers that have been retracted, please include the rationale for doing so in the manuscript text, or remove these references and replace them with relevant current references. Any changes to the reference list should be mentioned in the rebuttal letter that accompanies your revised manuscript. If you need to cite a retracted article, indicate the article’s retracted status in the References list and also include a citation and full reference for the retraction notice.

**Response:** Ok thank you for the suggestion. We have reviewed our reference and corrected as suggested above. As our study is systematic review and meta-analysis to minimize publication bias we have included primary studies which are not published. So it seems retracted articles but not. To solve this we have indicated the URL of the web page in which the studies were available. We have put the URL for reference 27, 28, 30, 41, 43 as they were not published but available at research repositories and other webpages and indicate reference 38 as it was a thesis. Additionally reference 20 and 45 were not correctly cited in the previous manuscript version and we have correctly cited those references.
