# Supplementary material for: Determinants of evidence-based practice among health care professionals in Ethiopia: A systematic review and meta-analysis
Source: PLoS One. 2023 Nov 9;18(11):e0293902. doi: 10.1371/journal.pone.0293902 (PMC10635493; doi:10.1371/journal.pone.0293902)
Supplement: Attachment — Submitted filename: Response to Reviewers.3rd.docx [file pone.0293902.s006.docx]

**A point-by-point response to reviewers and editor**

First of all, we would like to say thank you to both the reviewers and editor, we are so lucky to have you who had a great contribution to the improvement of this work. Saying this we have addressed the given comments as follows.

**Journal requirements**

Please review your reference list to ensure that it is complete and correct. If you have cited papers that have been retracted, please include the rationale for doing so in the manuscript text, or remove these references and replace them with relevant current references. Any changes to the reference list should be mentioned in the rebuttal letter that accompanies your revised manuscript. If you need to cite a retracted article, indicate the article’s retracted status in the References list and also include a citation and full reference for the retraction notice.

**Response:** Ok thank you for the suggestion. This suggestion was given previously and we have addressed it and submitted our response and revised version. As we have suggested we have reviewed our reference and corrected. As our study is systematic review and meta-analysis to minimize publication bias we have included primary studies which are not published. So it seems retracted articles but not. To solve this we have indicated the URL of the web page in which the studies were available. We have put the URL for reference 27, 28, 30, 41, 43 as they were not published but available at research repositories and other webpages and indicate reference 38 as it was a thesis. Additionally reference 20 and 45 were not correctly cited in the previous manuscript version and we have correctly cited those references.

**Reviewer # 3**

**Comment:** Table 1: Show more characteristics of studies, such as age, sex and etc.

**Response:** Ok thank you for your comment. We have tried to extract other characteristics of study participants of included primary studies but some studies missed and others may report and also the categories might not be the same across the included primary studies. For example the age category of study participants in the primary studies is not uniform so to present it in table form as Table 1not feasible. So we have tried to include important characteristics of primary studies that are linked with the study outcome and common for all included studies.

**Comment:** It should be “sample size” instead of “sample”.

**Response:** Ok thank you for your comment. We accept the reviewer comment and correct it as the reviewer suggested as shown in the track change in the revised manuscript.

**Comment:** How do you define quality of study as “good”?

**Response:** Ok thank you for the issue raised. The quality of included studies is evaluated using the Newcastle Ottawa Quality Assessment scale score. According to the scale grading if the sum of scale score is greater than or equal to 7 the study is classified as good and if below 7 it is graded as poor. In our case, the scale score of all included primary studies lies from 8 to 9 which is good. However we have not explained how we graded the quality of included studies in the introductory paragraph that is our mistake. We accept the review comment and explain how we grade the quality of included studies in the revised manuscript as shown in track change.

**Comment:** Table 2: Different studies may include different covariates in logistic regression. Simply pooling the adjusted odds ratios coming from different regression models may cause bias. List the details of the regression from each study to evaluate whether those models are comparable.

**Response:** Ok thank you for the issue raised. In identification of determinants of EBP utilization we have used factors which are associated in two or more primary studies which report adjusted odd ratio (AOR) which are output of multivariable logistic regression. All adjusted odd ratio (AOR) of primary studies which we have used in identification the overall determinants of EBP are output of multivariable logistic regression. However we have not describe which regression model output we have used and the reviewer concern is right and we accept the reviewer suggestion and make it clear in the revised manuscript as shown in the track change.

**Comment:** Page 6: better say “The secondary outcomes were factors associated with the evidence based practice of health professionals.”

**Response:** ok thank you for your comment. We accept your comment and rewrite it as shown in the track change of the revised manuscript.

**Comment:** Figure 5 is not necessary and can be omitted.

**Response:** ok thank you for your comment. However Figure 5 is about sensitivity analysis result of our review which shows whether single study dominated the overall pooled prevalence of EBP utilization of health professional or not. Unless it is graphically presented and explained there is no statistical test which prove the sensitivity analysis of our review; so we have decided to present figure 5 as graphical explanation of sensitivity analysis.

**Comment:** Flowchart: list each reason and N of exclusion. It is not clear to state “Exclude by title or by reading abstract”

**Response:** ok thank you for your comment. We have used the updated Preferred Reporting Items for Systematic Review and Meta-Analysis flow chart. In the flow chart exclusion by title and abstract has no reason because it is made before assessment by eligibility criteria. The reason for exclusion by title and by abstract is made if the title and abstract of the article is not complimentary with the current topic under review it would be excluded. Reason for exclusion is listed in the step of screening after full text articles are evaluated against the eligibility criteria then the number of article excluded and the respective reason for exclusion is explained. So we have explained the reason for the exclusion of 25 articles as the outcome of interest is not reported.

**Comment:** Conclusion: when you say the prevalence of EBP was low, are you comparing it to other African countries or western countries?

**Response:** ok thank you for the issue raised. As we know the conclusion of a scientific paper is written after we compare and contrast our finding with the existing finding by others. Conclusion is judgmental summarization of the current finding with in light of the existed evidence and expected standard. So in our review what we have done is we summarizes our finding / the prevalence of good EBP/ by comparing it with other finding that we made in discussion section and with the expected level of EBP utilization. Since conclusion summarize all contents of a scientific paper we are aiming to include the points in the discussion too. We understand the reviewer concern and try to make the conclusion more informative and clear as shown in track change in the revised manuscript. Once again thank you very much.
