# Supplementary material for: Determinants of evidence-based practice among health care professionals in Ethiopia: A systematic review and meta-analysis
Source: PLoS One. 2023 Nov 9;18(11):e0293902. doi: 10.1371/journal.pone.0293902 (PMC10635493; doi:10.1371/journal.pone.0293902)
Supplement: Attachment — Submitted filename: Response to Reviewers.4th.docx [file pone.0293902.s007.docx]

**A point-by-point response to reviewers and editor**

First of all, we would like to say thank you to both the reviewers and editor, we are so lucky to have you who had a great contribution to the improvement of this work. Saying this we have addressed the given comments as follows.

**Journal requirements**

Please review your reference list to ensure that it is complete and correct. If you have cited papers that have been retracted, please include the rationale for doing so in the manuscript text, or remove these references and replace them with relevant current references. Any changes to the reference list should be mentioned in the rebuttal letter that accompanies your revised manuscript. If you need to cite a retracted article, indicate the article’s retracted status in the References list and also include a citation and full reference for the retraction notice.

**Response:** Ok thank you for the suggestion. This suggestion was given previously and we have addressed it and submitted our response in the revised version. As we have suggested we have reviewed our reference and corrected. As our study is systematic review and meta-analysis to minimize publication bias we have included primary studies which are not published. So it seems retracted articles but not. To solve this we have indicated the URL of the web page in which the studies were available.

**ACADEMIC EDITOR Comments**

**Comment:** Topics-needs modification (which health care profession? Nurse, medical professional, midwifery). No need mixing these health professionals in one

**Response:** Ok thank you for your comment. Our article is a systematic review and meta-analysis which reviews primary studies and synthesize evidence. So those primary studies included in our review were conducted among health care professionals including nurses, medical professionals, midwifes and other health professionals together as study participant in each primary studies. That means one primary study include nurses, medical doctors, midwifes and other health professionals together as study participant. So it was limitation of those included primary studies. While we do the meta-analysis we are unable to specify to specific professionals since we cannot get the level of evidence based practice of those specific professionals because those the primary studies has gross level of evidence based practice of the health professional as a whole. Even if it was limitation of primary studies, we understand the editor concern and try to put it as limitation of our review as shown in highlighted text in the revised manuscript.

**Comment:** Quality assessment -Add quality assessment of the included article as supplement files

**Response:** Ok thank you for your comment. We have added the modified Newcastle Ottawa Quality Assessment Scale for cross-sectional studies as supplementary file labeled as **S2 Table** and also cited in the manuscript in line 143.

**Comment:** How to calculated pooled AOR using AOR of each study?

**Response:** Ok thank you for your question. Pooled AOR can be calculated using two methods. The first is using AOR of primary studies; second using number of events and number of subjects in each group. In our review we have used the first method sometimes known as the conventional method. In this method we extract the point estimate of AOR, the upper and lower confidence intervals of AOR of each studies for respective variable. Then the logarithm of those extracted indices is generated using STATA. Then the logarithm of those extracted indices used as effect size in each study, along with an estimate of its variance. Those estimated variances are usually assumed to be equal to the true variances. Finally using the metan command we have generated the pooled adjusted odd ratio on STATA 17.

**Reviewer # 3**

**Comment:** Reviewer #3: I thought the results of Figure 5 can be explained in the text. If the authors insist to add figure 5, please add more details about what the figure is presenting and what the interpretation is for general audience.

**Response:** Ok thank you for your comment. We accept the reviewer suggestion and omit figure 5 and explain the result of the figure by the text in the revised version of the manuscript.

Once again we want to say thank you very much.
